# A correlation study of sustainable development goal (SDG) interactions

**DOI:** 10.1007/s11135-022-01443-4

**Published:** 2022-06-13

**Authors:** Sheeba Pakkan, Christopher Sudhakar, Shubham Tripathi, Mahabaleshwara Rao

**Affiliations:** 1 Manipal Academy of Higher Education, Manipal, Karnataka India; 2 Department of Quality, Manipal Academy of Higher Education, Manipal, Karnataka India; 3 Department of Library and Information Sciences, Manipal Academy of Higher Education, Manipal, Karnataka India

**Keywords:** Sustainable development goal, Impact ranking, Correlation, Exploratory data analysis, Future prospect, Research policy

## Abstract

As universities are the change agent of society, institutions from all nations set their goals to transform the world by exploring various societal challenges that humans are facing. Together, the higher education systems across the world developing strategies based on the United Nations’ Sustainable Development Goals (SDGs). The current study aimed to provide policymakers, academics, and researchers an insight on the influence of 16 SDGs on each other paving the way for the universities to set a clear goal in attaining Sustainable Development goals by 2030. To analyze the SDGs’ interactions towards each other, 201,844 research publications from India during five years on 16 SDGs are retrieved from the Scopus database. Spearman Rank Correlation is applied to understand the correlation of each SDG towards one another. We could observe converging results out of the interactions among the SDGs. A significant positive and moderately positive correlation between pairs of SDGs are identified. While a significant number of negative correlations is also classified which need deep thinking among researchers to develop healthy relationships. The most frequent interactions between SDGs is a positive sign for any university in strategizing the goal towards SDGs. The association of all university stakeholders and some constitutional and cultural changes are necessary to put SDGs at the core of the management of the university. Embracing this task by researchers will improve the overall performance of universities. The analysis presented in the present study is useful for academics, governments, funding agencies, researchers, and policy-makers.

## Introduction

United Nations Sustainable Development Goals (SDGs) consist of 17 goals, 169 constituent targets, and 230 indicators, an evidence-based indicator, which is to transform the whole world into a sustainable one. The SDGs strategically allow universities/institutions to monitor and gauge their research activities strategies and publish the outcomes globally. This demands global sustainability benchmarking that applies across all universities in a national and global context (Sullivan et al. 2018). Times Higher Education Impact ranking gives universities ample opportunities to showcase their commitment towards society. Being the change leaders in education, research, and innovation, universities have a crucial role in developing a sustainable community. The new generation universities are more diverse in structure and are more focused on societal needs and benefits, as described by (Vilalta et al. 2018).

The sustainable development goals start from no poverty, touching all significant aspects of global concern like gender quality, sustainable economic growth, environmental preservation, climate action, and good health by addressing all countries (Vilalta et al. 2018). SDGs signify the global civic ambitions and for the educational institutions; it is a noble effort to address the important societal issues. SDGs represent an elaborate plan for the universities to achieve progressive societal changes (Jain et al. 2019). The inter-connectedness among these SDG goals renders planning, implementation, and monitoring challenges as far as university research is concerned. The extraordinary demand is executed by SDGs on the national statistical systems demands to generate and analyze an exceptional quantity of data. It is becoming a vast and complex issue as a whole (Jain et al. 2019).

Times Higher Education launched its SDG ranking in 2019 in the name of THE Impact Ranking (Barrick et al. 2019). Universities around the world are engaging to reach the SDGs with the world’s ambition to achieve the SDGs, especially on poverty (SDG1), livelihood and food (SDG2), health (SDG3), education (SDG4), employment (SDG5), and economic growth, infrastructure (SDG9,10) (Stephens et al. 2008). This article attempts to quantify the SDG’s contribution to BRICS countries for the years 2015–19. Detailed analysis is done taking Indian contribution on 16 SDGs and attempted to find out the correlation between each SDG to the other. (Kapur et al. 2016; Ioannidis et al. 2007) also did a critical study on different aspects of ranking in detail. This research reveals how a university can manage its research contributions for the benefit of their society by linking the research with clearly defined SDGs. The work is relevant because the Times Higher Education impact ranking measures the research contribution of an institution based on16 SDGs with clearly defined metrics. In this study, we have tried to find the relationship between 16 SDGs and the correlation of each other. This is important for a university as they are the change agent who wants to dedicate their research output to the development of society (Molinari et al. 2008). Any university promoting research can follow this finding as a guide and try to interconnect each SDG to the related one while planning their thrust area of study and applying for research grants. It will be a good start for any researcher to deeply evaluate research objectives and link the work with all relevant SDGs possible to get a positive impact on their research.

## Literature review

The announcement of 22nd April 2020 Times Higher Education Impact Ranking has clearly shown where each country stands in promoting SDGs. Universities play an essential role in building a knowledgeable society and thereby help in building a sustainable and secure future for the society (SDG summit 2015). In September 2015, the United Nations (UN) adopted the 2030 Agenda for Sustainable Development (Rosen et al. 2020). With one goal of having a sustainable society, the whole world is working towards achieving the 17 SDG targets. (Vilalta et al. 2018) also mentioned in their study that universities are no longer act as a political instrument of social policy but progressively an integral part of building a sustainable society. Universities are the primary contributors to building a sustainable society; the scholarly output coming from the Universities is of more importance in the present scenario, as indicated by (Stephens et al. 2008 and Fonseca et al. 2020).

Several pieces of literature (Sullivan et al. 2018; Vilalta et al. 2018; Barrick et al. 2019; Jain et al.^2019^). Which studied the different aspects of SDGs and the challenges and opportunities of different themes of SDGs in achieving several targets. The contribution of universities has to be focused on all 16 SDGs. This requires skills and mindsets to contribute and meet these challenges (Perovi’c et al. 2020; Fuso-Nerini et al. 2017). Universities’ importance and obligation to sustain sustainability will lead to SDG inclusion in the policies, and together, aims to achieve the set goals are well appreciated. As (Rosen et al.^2020^) explained in the research article about the universities’ contribution and the starting of Impact ranking to measure it towards 16 SDGs. Approaching the impact ranking requires skills and mindset to contribute and meet the SDG’s challenges. As change agents and creators of opportunities, universities need to undergo different perspectives and expectations to maintain sustainability. It will lead to SDG inclusion in future planning to achieve the set goals (Stephens et al. 2008; Ivanova et al. 2016).

The unexpected challenges faced by society due to Covid-19 infectious disease adversely affected the current trends and patterns of resource use, improvement in health care, and research in these areas. Research article mentioning the correlation between SDGs and their necessity while doing a related study is still not accepted or re-searched in their full strength to the research world. (Fonseca, L. M. et al. 2020) an elaborated correlation study was done, which explains the importance of correlation of SDGs among each other and the areas (SDGs) which need to be carefully dealt with while doing research. This is because improving research in one area should not adversely affect the other areas. (Molinari et al. 2008; Fuso-Nerini et al. 2017). Here intensifies the importance of SDG 17 Partnerships for the Goals. Research in these areas is of great relevance at the present stage. The pandemic has challenged the health sector research, lively-hood, poverty, education in rural villages, and economic growth. This is a challenging time for all universities worldwide to think of international investments and support to lead to innovative technological developments (Jain et al. 2019). Research-oriented institutions have ample opportunities to evaluate these challenges and recommend solutions for them (Nilsson et al. 2016; Singha et al. 2018; Pradhan 2017a, b).

## Methodology

The data for the study related to 16 SDGs has been retrieved from the Scopus database. Properly defined keywords used in Scopus have retrieved data pertaining to particular SDGs. We have quantified the research publications of the world on 16 SDGs and also quantified the research contributions of the BRICS countries to benchmark with each other’s contribution towards achieving the sustainability of the world. The period selected for data retrieval is from 2015 to 19. A detailed analysis of the publication contribution of India on each SDG is done using Spearman’s Rank Correlation. Tableau software is used for visualizing the analyzed data. The nonparametric Spearman’s Rank Correlation (ρ) analysis is carried out in this study to know the relationship between each SDG (Spearman 1987). Here, the data is nonlinearly correlated between the variables so Spearman’s analysis is the best choice as it is less sensitive to outliers (Pearson’s 2011). We have performed the correlation analysis with the research data from India for the past five years (2015–19) on 16 SDGs. This result will explain the synergies/trade-offs in the SDGs and it will help the institutions to plan their future.

The present work aims at knowing the contribution of BRICS nations in strengthening the SDGs in the country and thereby enhancing cooperation and establishing networks between universities in the world for research and education. In addition, it aims to improve the interactions between University research, its contribution to societal improvement, and strategy in formulating new research policy. The work finally concludes by finding out the correlation between each SDG and its contributions to one another. The main objective of the present work is to explore all the 16 SDGs research contributions and their relationship with each other. The percentage of relationships is explained using correlation metrics. This will be the aspirational guidance for the 2020–2030 period to strengthen the relationship more.

### Terminologies and explanations

- SDG (Sustainable Development Goals (Table 1)): United Nations Sustainable Development Goals consist of 17 goals, 169 constituent targets, and 230 indicators built to transform the whole world into a sustainable one. SDG 17 Partnerships for the Goals is not considered for the study as the target for this is achieved from all other 16 SDGs.
- Scholarly Output: The research article published by a researcher or researchers.
- BRICS: The BRICS countries are Brazil, Russia, India, China, and South Africa.

**Table 1:** SDGs and abbreviations

Sustainable Development Goals (SDGs)	
SDG 01. No poverty	SDG 09. Industry, innovation, and infrastructure
SDG 02. Zero hunger	SDG 10. Reduced inequalities
SDG 03. Good health and well-being	SDG 11. Sustainable cities and communities
SDG 04. Quality education	SDG 12. Responsible consumption and production
SDG 05. Gender equality	SDG 13. Climate action
SDG 06. Clean water and sanitation	SDG 14. Life below water
SDG 07. Affordable and clean energy	SDG 15. Life on land
SDG 08. Decent work and economic Growth	SDG 16. Peace, justice and strong institutions

### Materials and methods

With the overall objective to identify the correlation between 16 SDGs with each other related to the focus areas of academia in India, the study was designed to get the source of the data from SciVal and Scopus. According to (Fonseca et al. 2020) correlation analysis is the best method to map the relationship between different variables. The search method used was keyword search, there are a set of keywords for each SDG, which is developed by subject experts to map the scientific publications in the Scopus database. For example for SDG 3- Good Health and Well-Being”, the keyword is formulated as: TITLE-ABS-KEY ( ( ( human AND ( health* OR disease* OR illness* OR medicine* OR mortality)) OR {battered child syndrome} OR {cardiovascular disease} OR {cardiovascular diseases} OR {chagas} OR {child abuse} OR {child neglect} OR {child well-being index} OR {youth well-being index} OR {child wellbeing index} OR {youth wellbeing index} OR {water-borne disease} OR {water-borne diseases} OR {water borne disease} OR {water borne diseases} OR {tropical disease} OR {tropical diseases} OR {chronic respiratory disease} OR {chronic respiratory diseases} OR {infectious disease} OR {infectious diseases} OR {sexually-transmitted disease} OR {sexually transmitted disease} OR {sexually-transmitted diseases} OR {sexually transmitted diseases} OR {communicable disease} OR {communicable diseases} OR aids OR hiv OR {human immunodeficiency virus} OR tuberculosis OR malaria OR hepatitis OR polio* OR vaccin* OR cancer* OR diabet* OR {maternal mortality} OR {child mortality} OR {childbirth complications} OR {neonatal mortality} OR {neo-natal mortality} OR {premature mortality} OR {infant mortality} OR {quality adjusted life year} OR {maternal health} OR {preventable death} OR {preventable deaths} OR {tobacco control} OR {substance abuse} OR {drug abuse} OR {tobacco use} OR {alcohol use} OR {substance addiction} OR {drug addiction} OR {tobacco addiction} OR alcoholism OR suicid* OR {postnatal depression} OR {post-natal depression} OR {zika virus} OR dengue OR schistosomiasis OR {sleeping sickness} OR ebola OR {mental health} OR {mental disorder} OR {mental illness} OR {mental illnesses} OR {measles} OR {neglected disease} OR {neglected diseases} OR diarrhea OR diarrhoea OR cholera OR dysentery OR {typhoid fever} OR {traffic accident} OR {traffic accidents} OR {healthy lifestyle} OR {life expectancy} OR {life expectancies} OR {health policy} OR ( {health system} AND ( access OR accessible)) OR {health risk} OR {health risks} OR {inclusive health} OR obesity OR {social determinants of health} OR {psychological harm} OR {psychological wellbeing} OR {psychological well-being} OR {psychological well being} OR {public health})). The data retrieved for BRICS countries from SciVal “Trends” module. Selection of each SDG is the first step followed by year range fixed 2015 to 19. “Trends” module have the provision to select “Countries & Regions” and narrow down the search to the specific country you decided to select.

For the correlation study, the data is retrieved from Scopus using the appropriate keywords. A detailed study on 16 SDGs has been done retrieving the data for the country India and its SDG contributions. The period chosen for the study was a five-year window (2015–2019). “Title” of the article is collected for 16 SDGs. The title is matched using python programming and the count of matches is calculated for each SDG. Due to the different theoretical ramifications of SDGs, it is important to transfigure the text data into a measurable gauge to study the impact. Spearman’s Rank Correlation metrics are built with an overall aim to identify SDG-related goals and relationships in particular with each other. Min–Max scaling is used to normalize the data (Spearman 1987). The principle scores were normalized to the same scale (0–1). Python’s Scikit Learn library was used for this process.

### Data extraction and preprocessing

The data for analysis has been retrieved from Scopus, where row data sets of bibliographic details like; links to articles, affiliation, Scopus author IDs, author names, etc., were available. Natural Language Processing (NLP) technology is used to clean the data and make it in the desired form.

## Total publication on 16 SDGS in the world

**Fig. 1:**
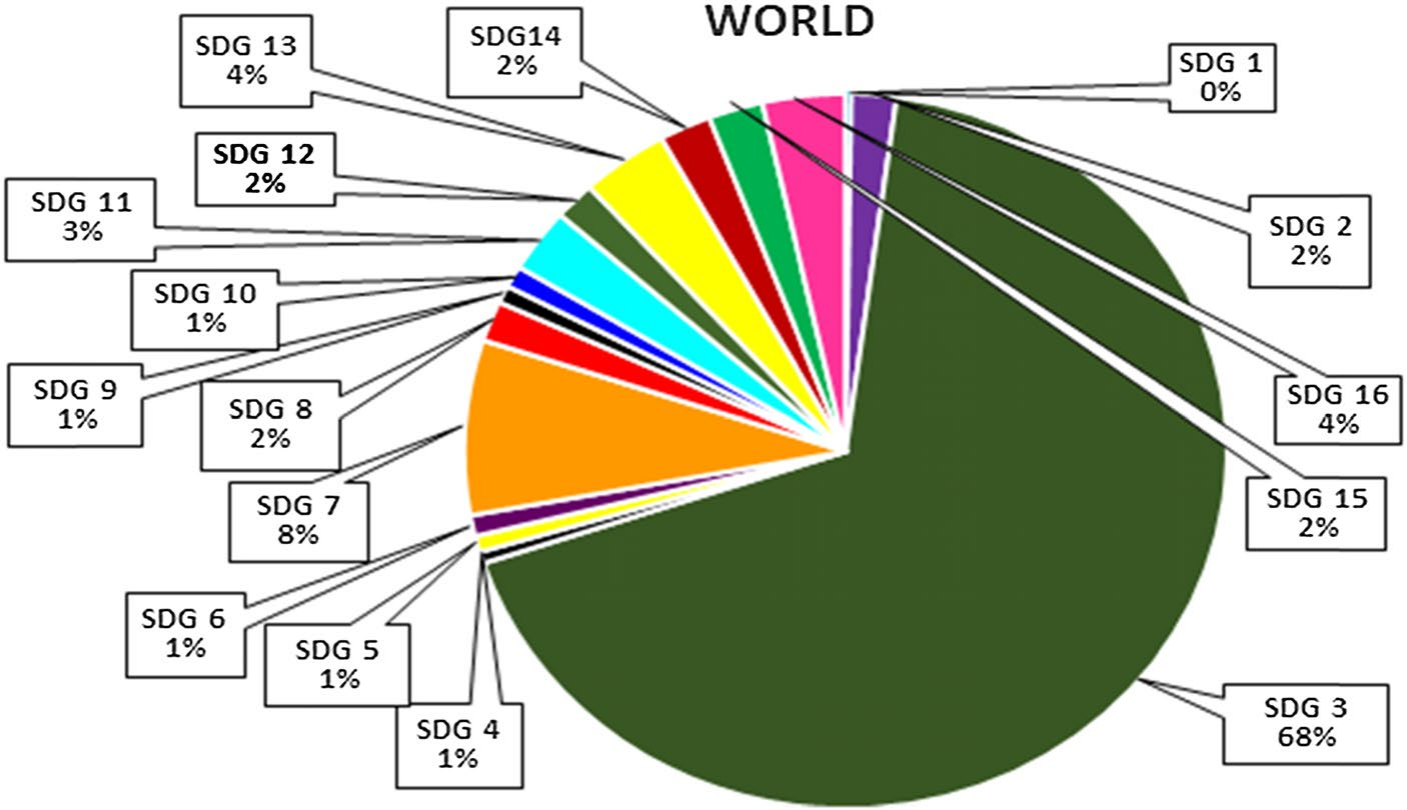
Among the total world publications the percentage distribution of all the 16 SDGs

Different metrics are considered for the analysis, and the sample data is taken for five years duration (2015–19). The overall SDG contribution of the world is 43, 86,588 and the distribution percentages for 16 SDGs are plotted in Fig. 1. Among the total publications of the world for the duration of five years, publications related to16 SDG were only considered for the study. The data were plotted using the python graphics library Plotly. The highest number of publications (3 081 203) comes from SDG 3 Good Health and Well-being followed by SDG 7, Affordable and Clean Energy having a total publication number of 3 589 30. The lowest quantity of world publications contribution goes to SDG 1 No Poverty with only 10,533 publications, followed by SDG 4 Quality Education having a count of 23,806. It is quite natural that SDG 1 No poverty and SDG 4 Quality Education are the vital component of SDG 3 Good Health and Well-being, and it is surprising to see that it does not keep any relation between them as publications are concerned.

## The publication contribution of brics nations on 16 SDGs

We have analyzed the contribution of BRICS countries (Brazil, Russia, India, China, and South Africa) to see the number of publications they contribute to the UN SDGs (Table 2). Worlds’ largest contributions come from SDG 3–Good Health and Well-being followed by SDG 7–Affordable and Clean Energy, as far as BRICS countries are concerned they are also contributing more to these two SDGs. China is the most significant contributor among BRICS countries. The scholarly output of India is more than China in SDG 5, Gender Equality and SDG 16, Peace and Justice Strong Institutions. When evaluating SDG 5, Gender Equality, after India, the countries South Africa and Brazil contributed more than China. To keep a strong stand in the world university impact ranking, Indian Universities have to work more intensively to focus their research in the areas defined by UN SDGs. Most of the 16 SDGs are inter-connected with each other, and careful management of research programs on these focused areas can improve Indian Universities’ ranking status in the world

**Table 2:** Total publication number for 16 SDGs contributed by BRICS Countries and the World

	SDG1	SDG2	SDG3	SDG4	SDG5	SDG6	SDG7	SDG8	SDG9	SDG10	SDG11	SDG12	SDG13	SDG14	SDG15	SDG16
Brazil	285	4505	80,641	1313	753	1457	7123	1721	906	946	4260	2719	4873	3638	4822	3108
Russia	146	1513	29,574	1016	196	412	7048	4175	1116	1060	2952	1400	3641	2657	2075	2881
India	613	6987	121,979	427	1125	2763	27,142	3621	2310	1182	7031	5125	7134	4213	4372	5820
China	627	14,775	372,391	978	436	9685	95,489	1134	6443	2408	27,509	14,843	25,852	15,053	20,162	5402
South Africa	518	1863	27,225	585	957	831	2573	2447	634	1048	1649	946	2770	1435	1878	3154
World	10,533	86,526	3,081,203	23,806	33,360	42,767	358,930	82,729	36,041	44,509	131,563	79,000	169,649	99,682	104,693	159,734

### Brics contribution on SDG 1, SDG 2, SDG 3, and SDG 4

The measure of the contribution of universities in any county on SDGs is a challenging one. The problem is how to quantify the publications in terms of SDGs when there is not much external information available. An option formulated in this quantification is the Impact Ranking by Times Higher Education. There are many limitations on ranking (Molinari et al. 2008) still, it is an eye-opener to the universities to act upon and formulate a proper strategy for a sustainable society.

**Fig. 2:**
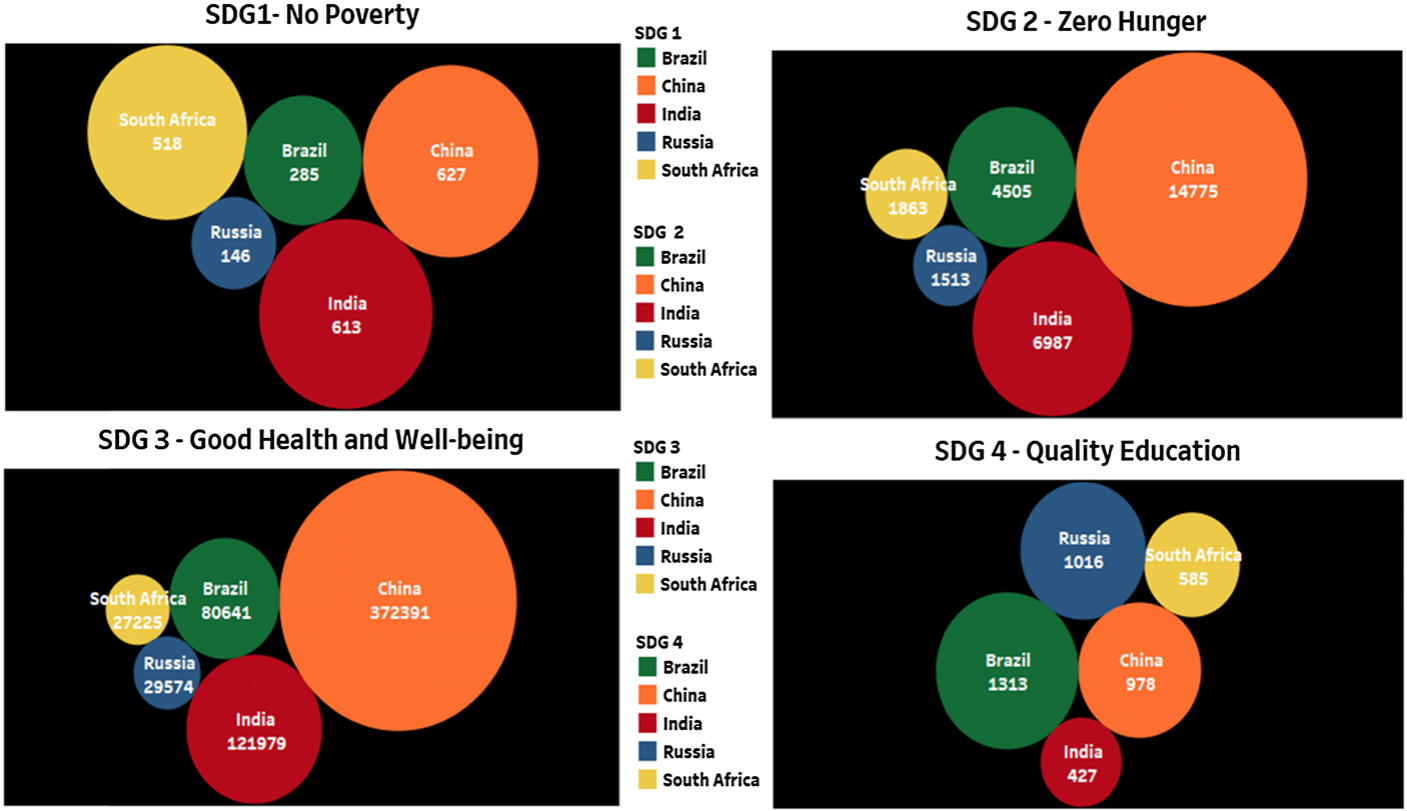
Publication contribution of BRICS countries on SDG 1, SDG 2, SDG 3, and SDG 4 for the year 2015–19

In Fig. 1, the core area in which more research is happening among BRICS countries is SDG 3–Good Health and Well-being. The quantity of publications is very less in the area SDG 1—No Poverty and it says that research in this area is less compared to other areas of importance. Among BRICS countries, Russia and Brazil contributed more in research publication number for SDG 4 (Quality Education) and India’s contribution comes less than those countries. The lack of strategies on gender equality and related commitment in recruiting and promoting women is reflected when we analyze the data on SDG 5 (Gender Equality). SDG 1 to SDG 5 in Fig. 2 is all some way or the other connected to each other. Research in one area can address many issues in the other areas too.

### Brics contribution ON SDG5, SDG6, SDG7, and SDG8

**Fig. 3:**
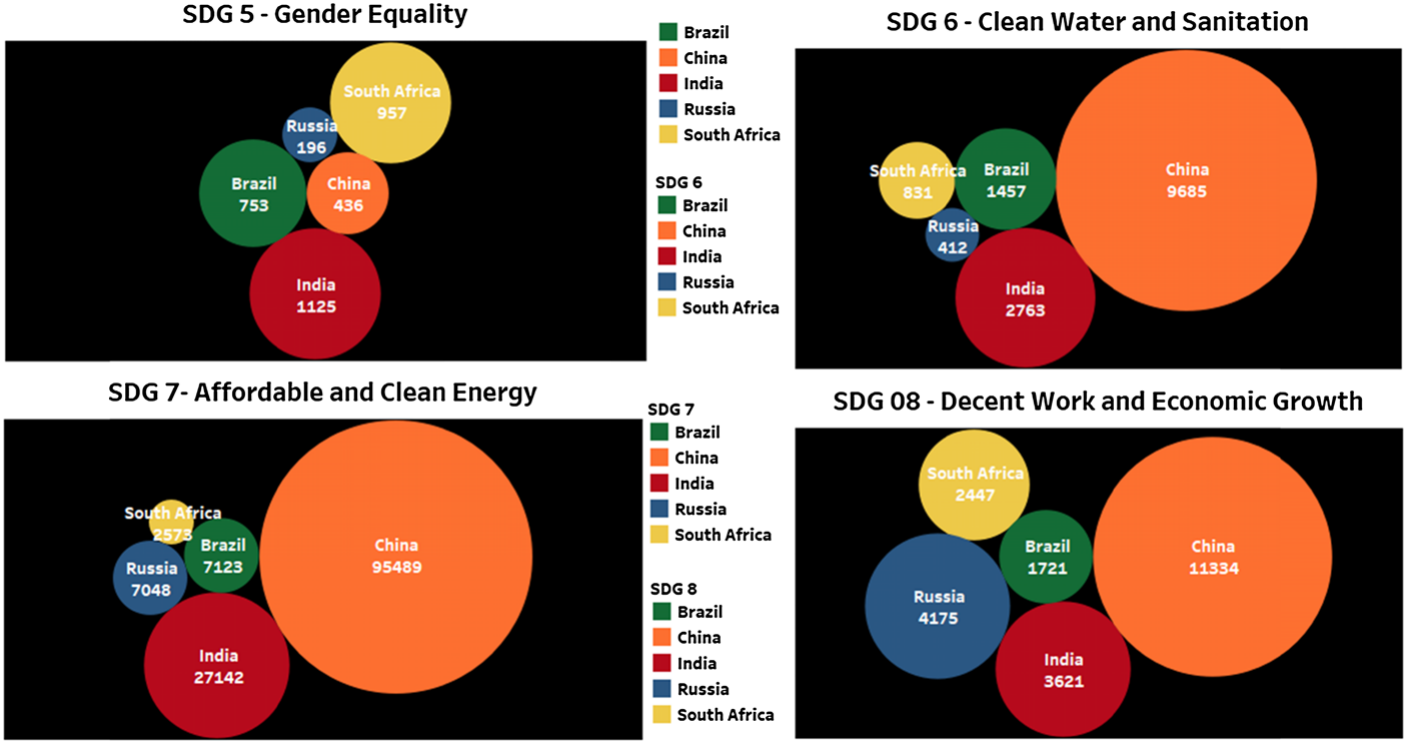
Publication contribution of BRICS countries on SDG5, SDG6, SDG7, and SDG8 for the year 2015–19

In Fig. 3, apart from SDG 5 Gender Equality, China leads in publication numbers. The rights of a citizen in research are reflected in SDG 5 and India leads in SDG 5 among BRICS countries. South Africa and Brazil contribute more than other countries.

This research area can be polished and more focused research can be nurtured in this area by other countries in BRICS. Among the sets in Fig. 3, SDG 5 is the only SDG where China is contributing less among the BRICS but intensive research is concentrated on energy production, consumption, and different aspects of it. China’s contribution to SDG 7 Affordable and Clean energy is visible in Fig. 3. It is very important to know the relationship between different areas of research. There must be a proper strategy to know, learn and include different related areas while doing proper research. A good extent of research is going on in SDG 7 by China more than other BRICS nations. India and other BRICS countries must make an effort to concentrate their research more on SDG 6, 7, and 8 like China, because of its relevance to the progress of any society.

### Brics contribution on SDG9, SDG10, SDG11, and SDG12

**Fig. 4:**
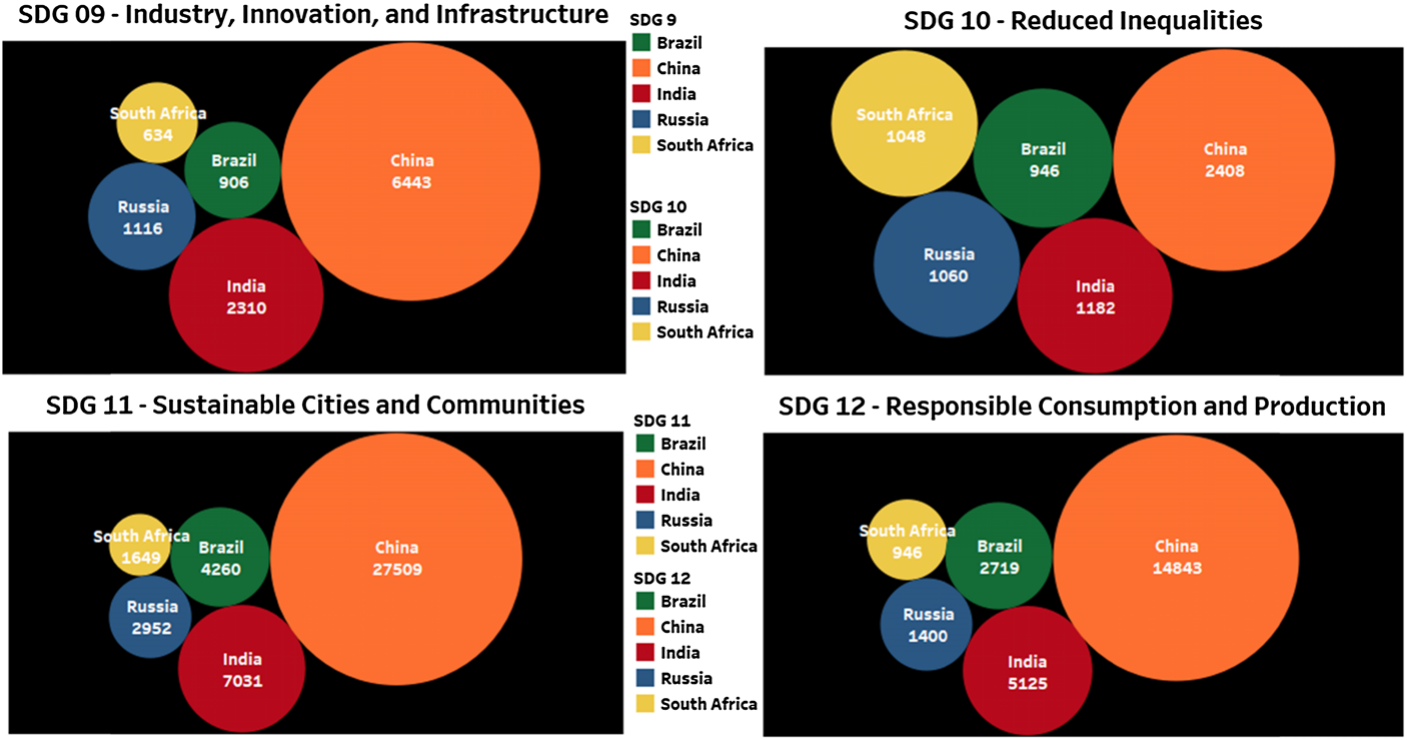
Publication contribution of BRICS countries on SDG9, SDG10, SDG11, and SDG12 for the year 2015–19

Figure 4 shows the BRICS contribution to SDG9, SDG10, SDG11, and SDG12. China is contributing more to SDGs 9, 10, 11, and 12. A close study on the data SDG 10 (Reduce Inequality), the publication contributions are almost equal in number among India, Russia, and South Africa. Here also China leads among all but the publication number shows that other countries can also achieve the target in time. In Fig. 4, SDG 11 and 12 are important SDGs that need more research and innovation to build a sustainable society. The contribution of universities will reflect in the form of publications that will be delivered to society in the form of technology transfer.

### Brics contribution on SDG 13, SDG 14, SDG 15, and SDG 16

**Fig. 5:**
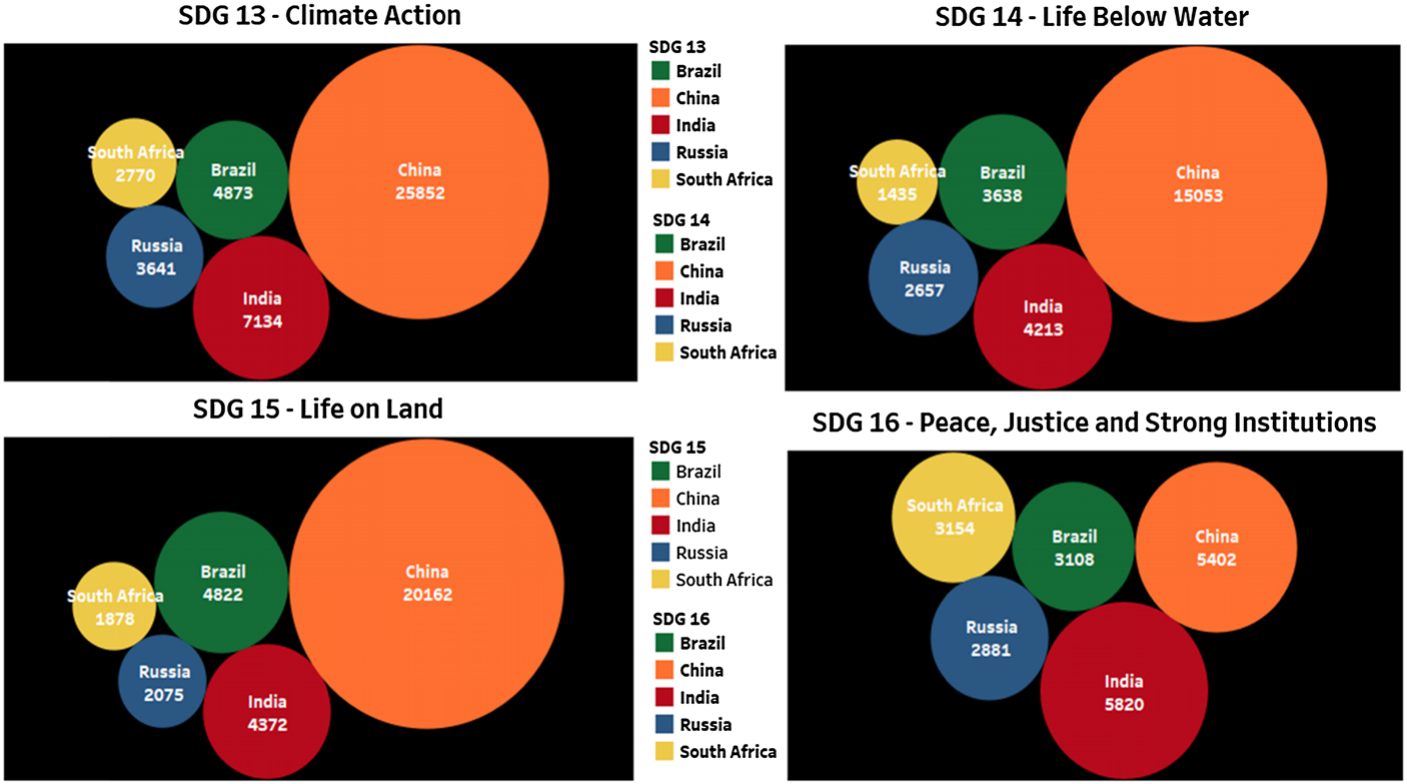
Publication contribution of BRICS countries on SDG13, SDG14, SDG15 and SDG16 for the year 2015–19

Indian research publication contribution to the important themes like SDG13 (Climate Action), SDG14 (Life Below Water), and SDG15 (Life on Land) (Fig. 5) is very less compared to the publications from China. This contrast of data may be the result of keywords used to express these SDGs in their publications. When we have a search by selecting the exact keywords, only the related documents will reflect in the result. Therefore, it is time for India to evaluate the keywords, which reflect each SDG and plan accordingly while publishing the related research work. While in SDG16 (Peace and Justice Strong Institutions), Indian research contributions are more than in China. It is high time for all universities in India to evaluate their publications, find out the reason for not reflecting its publications on 16 SDGs, and strategize a new policy for the mapping. University Grant Commission (UGC), Govt. of India can seriously look into all these aspects of SDGs metrics and give directions to universities to redirect research programs in tune with SDGs for global outreach.

## Correlation matrix of Indian contribution on 16 SDGs

The data for India on 16 SDGs was extracted using Scopus keyword search (Table 3). The dataset has been normalized to make it into a standard scale without disturbing the range. As mentioned earlier, Min–Max scaling is used to normalize the data. The complete nature of the SDGs indicates that a large number of potential publications across the 16 SDGs have to be considered by policymakers and an outline has to be proposed to illustrate SDG interactions. In this paper, we have done a systematic data-driven analysis of interactions between all SDG indicators. Statistically, we have tried to classify all 16 SDGs and their existing interactions with each other and classified them as synergies and trade-offs. In the present study, the progress in one SDG can be a goal favors to the progress in other SDGs and we can see a highly positive correlation among many SDGs. For example, while considering the data for SDG 2—Zero Hunger, the “Title” of the articles match with the “Titles” of all other SDGs one by one. It will generate data that matches both the SDGs. The more the number of “Titles” matches among SDGs will show the relationship of the SDG as stronger. While for the explanation of the negative correlation, we can say that even though there are fewer publications that talk about the relationship of both the SDGs the researchers have to put more effort and the academics has to develop new policies to strengthen the weak SDG bonding to be a stronger one.

**Table 3:** “Title” of all the 16 SDGs matched with each other and the matching data is in the table (data from Scopus for 2015–2019)

	SDG1	SDG2	SDG3	SDG4	SDG5	SDG6	SDG7	SDG8	SDG9	SDG10	SDG11	SDG12	SDG13	SDG14	SDG15	SDG16
SDG1	613	72	94	35	205	15	13	307	26	74	23	9	44	3	22	32
SDG2	72	6987	1055	6	35	216	210	170	20	417	187	265	801	68	533	30
SDG3	94	1055	124,891	92	391	491	462	131	102	158	114	537	493	250	273	962
SDG4	35	6	92	429	27	2	3	38	1	27	10	3	8	0	3	16
SDG5	205	35	391	27	1123	8	2	251	15	91	21	6	15	1	4	368
SDG6	15	216	491	2	8	2763	149	53	24	9	290	134	258	4	240	13
SDG7	13	210	462	3	2	149	27,049	360	162	57	664	731	745	46	124	102
SDG8	307	170	131	38	251	53	360	3621	498	233	208	252	195	118	120	124
SDG9	26	20	102	1	15	24	162	498	2303	29	436	130	49	23	25	27
SDG10	74	41	158	27	91	9	57	233	29	1182	55	13	36	5	14	79
SDG11	23	187	1144	10	21	290	664	208	436	55	7031	947	386	60	468	125
SDG12	9	265	537	3	6	134	731	252	130	13	947	5195	203	65	143	26
SDG13	44	801	493	8	15	258	745	195	49	36	386	203	7134	245	834	34
SDG14	3	68	250	0	1	46	118	23	10	5	60	65	245	4213	185	10
SDG15	22	533	273	3	4	240	124	23	25	14	468	143	834	185	4372	15
SDG16	32	30	982	16	368	13	102	124	27	79	125	26	34	10	15	5821

The current study was undertaken to assess and analyze the scientific publications on SDGs. The “Titles” of the articles will be selected and matched with other SDG articles “Titles”.. To explain it more clearly, I would like to take the example of SDG 1 having publication number 613 for India from 2015 to 2019 (Table 4). When the “Titles” matched with SDG 1 and SDG 2, one could see 72 publications from SDG 1 are reflected in SDG 2. The positive thing here is that the higher the relationship of the topics the more the correlation would be. If the researcher and the institutional administrators and policymakers look into the relationship of all SDG topics and the correlation metrics, it will be very easy for them to strategize their research accordingly. In the case of SDG 2, and SDG 1 even though the topics are related, and depend on each other (Zero Hunger and No Poverty) we could see only a few research works that talk about the relationship. The major concentration of the study is to give an insight to the researchers, academics, and policymakers on the correlation of SDGs. That can lead to strategizing suitable policies to implement and enrich SDG research in the country. We have used nonparametric Spearman’s Rank Correlation (ρ) in the present study, as the data is not normally distributed. To extract all possible combinations of the unique indicator data pairs for each SDG and to get the monotonic relationships, we have used Spearman’s Rank Correlation. Spearman’s Correlation Coefficient (ρ) provides a measure to evaluate the strength of an association between two variables. Spearman’s analysis can capture the nonlinear correlation between the variables and is less sensitive to outliers. Spearman’s analysis is widely used to identify general relations beyond the linear correlation between two variables in various disciplines (Spearman 1987).

**Table 4:**
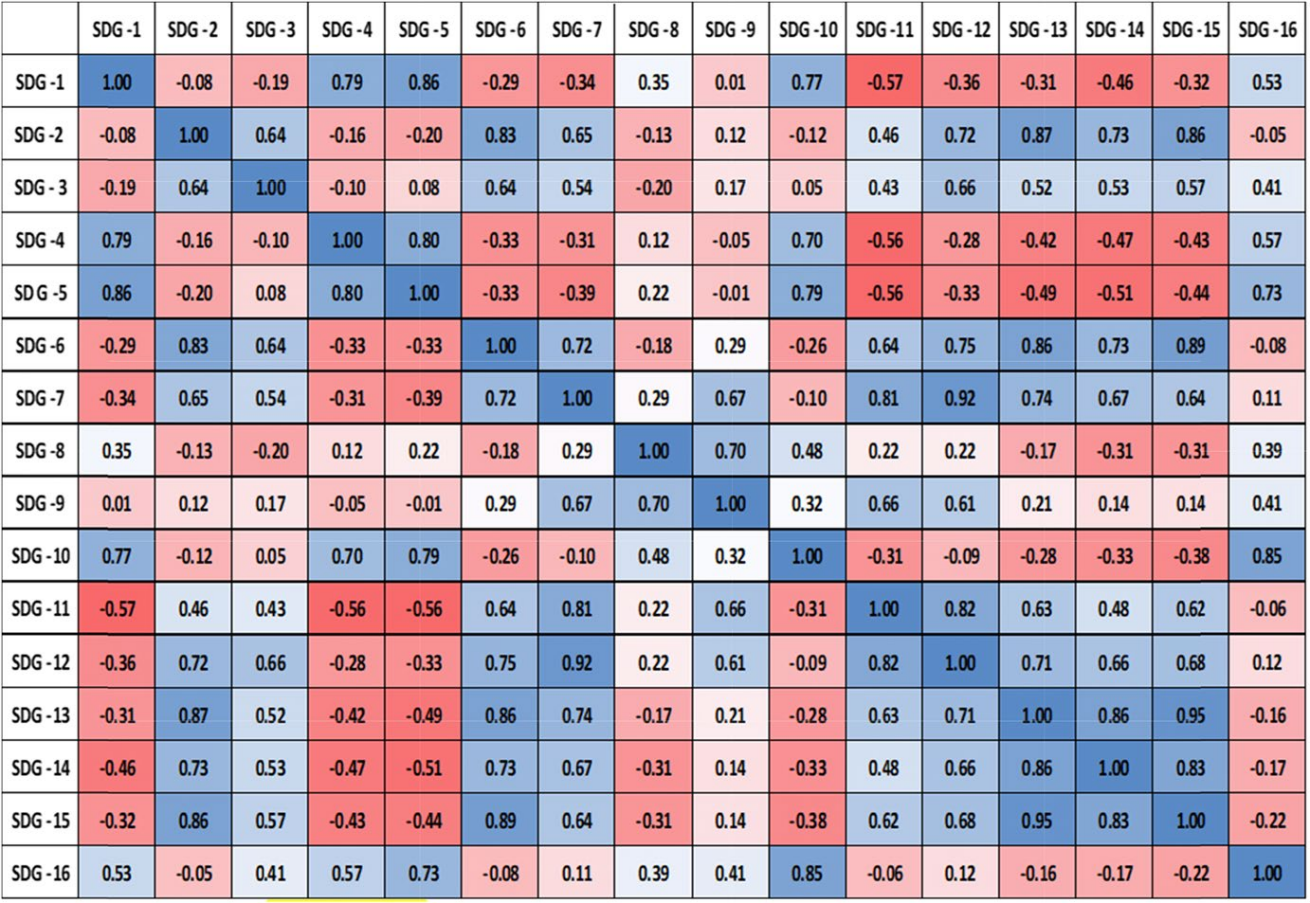
Correlation matrix of publications of India on 16 SDGs (data from Scopus for 2015–2019). (Color figure online)

Since Python has excellent support for statistical analysis, we built a correlation matrix using the Python programming language. Correlation analysis was carried out with the 16 Sustainable Development Goal datasets. We could find a high correlation among many SDGs. From our dataset of publication from India, the highly correlated variable in the SDG list keeping a threshold value of 0.8 and above is put in Table 5. Particularly, SDG1 (No poverty) with SDG5 (Gender Equality) the correlation is 0.86. SDG2 (Zero Hunger) with SDG6 (Clean Water and Sanitation), the correlation is 0.83. SDG13 (Climate Action) with SDG15 (Life on Land), the correlation is 0.95 and is highly correlated. It means there are 836 articles reflected in SDG 15 also. The research topic talks about climate action are also discussing life on land in their article. SDG6 with SDG13 (Climate Action) the correlation is 0.86. SDG 7—Affordable and Clean Energy with SDG12—Responsible Consumption and Production show synergetic relations with ρ values greater than 0.8. The SDGs which are highly correlated in the dataset are sharing the same document in both the related SDGs. A careful study of these highly correlated variables will help to plan clear strategies for a university. An institution that is enthusiastic to participate in the ranking process and wanted to strategize properly their future activities and get ready for world ranking can follow these studies as an example. Some publications are not mapped in any of the 16 SDGs whereas some are in the real sense related to one or the other SDGs. We should have a new strategy on each topic and can relate the same topic with highly correlated and moderately correlated SDGs. We have taken the data with a threshold value range from 0.5 to 0.79 and extracted the moderately correlated variables as shown in Table 6

**Table 5:**
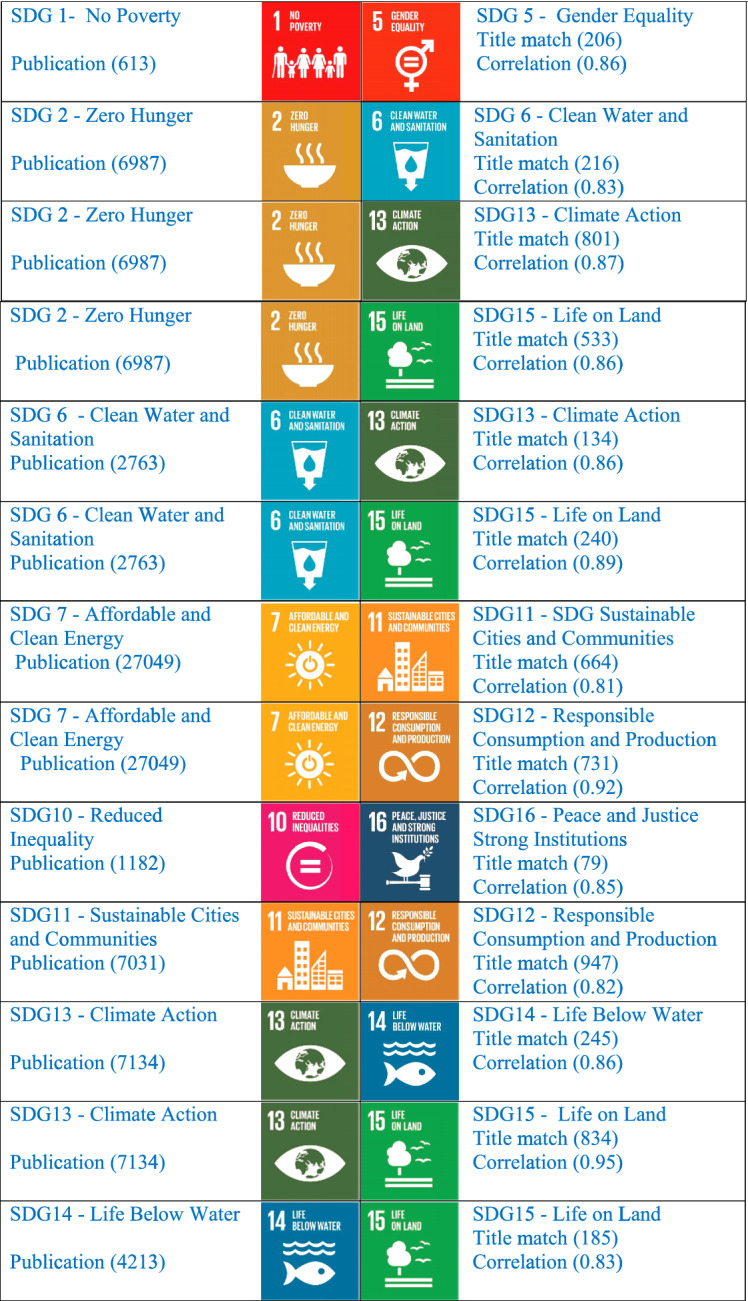
Highly correlated pairs of SDGs within the quantities (sets) of publication of India on 16 SDGs in Scopus for 2015–2019 (with threshold value 0.80 and above)

**Table 6:**
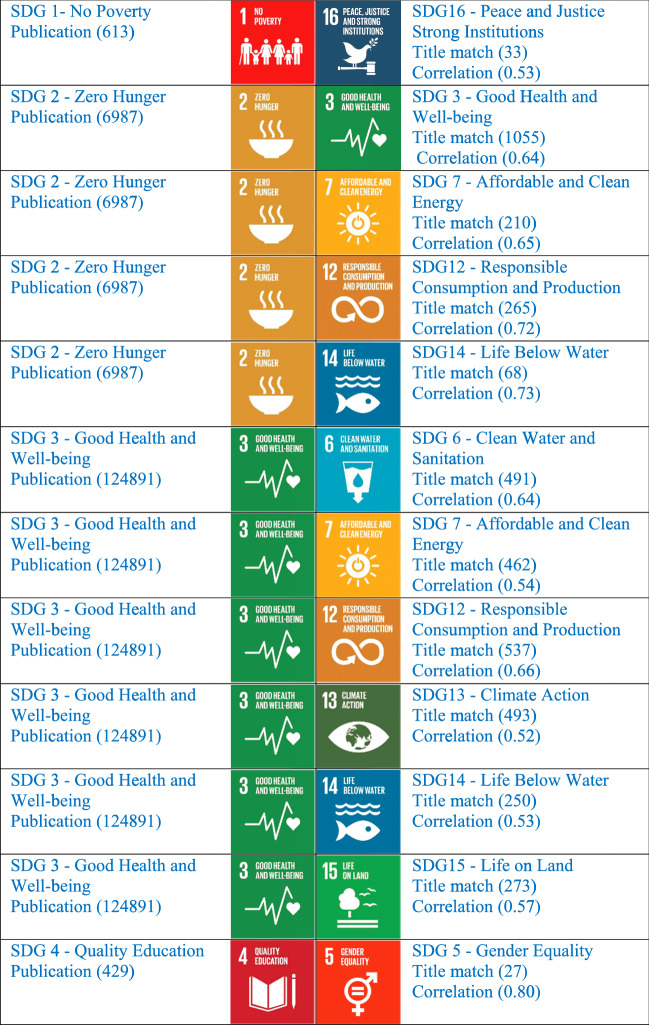

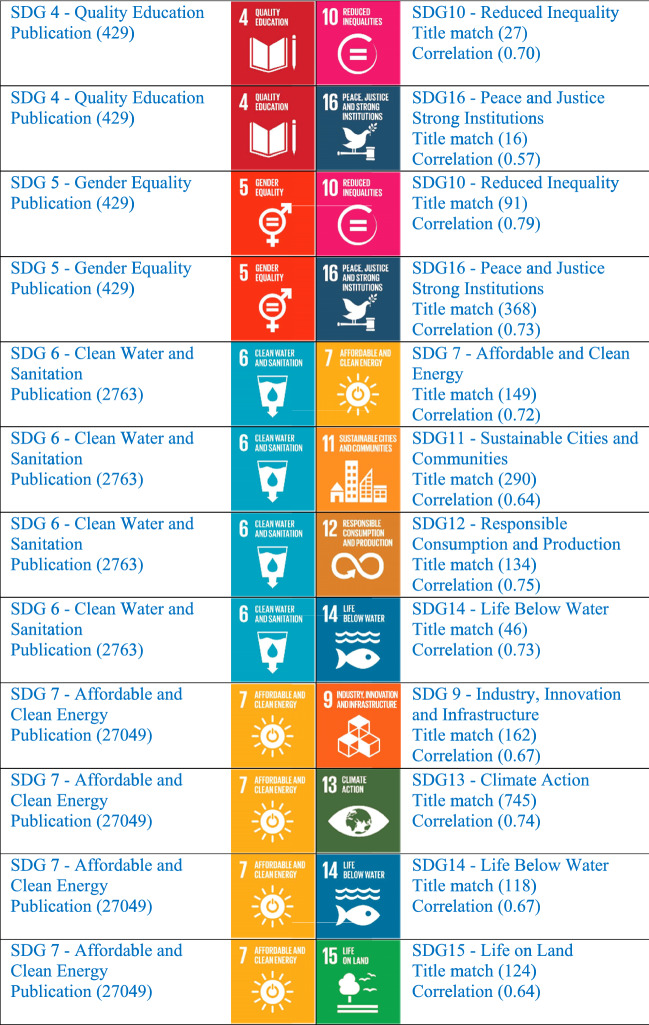

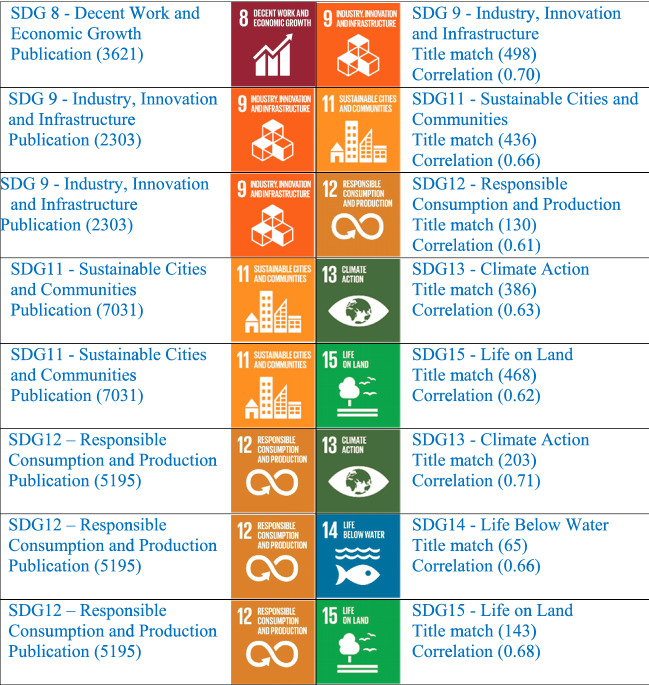
Moderately correlated pairs of SDGs within the quantities (sets) of publication of India on 16 SDGs in Scopus for 2015–2019 (with the threshold value 0.5 to 0.79)

Looking at the moderately correlated variables, it will be easy for any researcher and university to plan its publications in the related SDGs, so that with a limited number of publications you will be able to qualify in participating in different subject area rankings. The highly published area SDG 3 (Good Health and Well Being) is moderately correlated with SDG 2 (Zero Hunger) showing a correlation value of 0.64. It clearly shows that the need of the hour has come to an end for a better strategic evaluation of SDGs. It needs to have a study on the relationship of SDG publications and work on strengthening the SDG partnership more. If the institution can produce more papers in the same direction, by focusing on SDG3 and strengthening the relationship with all the related SDGs by doing related research and proper keywords, we can achieve a sustainable society very soon. The relationship between all 16 SDGs is worth studying and implementing. In reverse, it will give a better rank in the Times Higher Education Impact Ranking.

This study also highlights the existence of negative correlations between many SDGs and this is a matter to be considered seriously. Progress in one indicator must give an improvement in another indicator then we can expect drastic changes in the overall data mapping. In the present study, SDG1 (No Poverty) is negatively correlated with most of the SDGs like SDG 2,3,6,7,11,12,13,14 and 15. However, these research areas are mostly related to No poverty. If a university has a well-planned strategy in achieving the SDGs, it is very much necessary to study the collaboration of each SDG with one another and couple the publications, and promote them within their domain of influence. A university’s research and innovation always have a key role in helping the society where it belongs by addressing these challenges. Our analysis reveals that a well-planned strategic approach to SDG mapping will address almost all challenges in the community which in turn will help universities to address THE Impact Ranking. Our study highlights the existence of typically more interactions within and among the SDGs. This specifies a strong groundwork for the successful implementation of the SDG indicators in future research. The evidence calls for a deeper investigation and demands advanced strategic planning. All related research work needs to act as a system of interacting cogwheels that together move with different SDGs. Therefore, policies promoting cross-sectoral and supportive SDG relations have to be instigated. It will play a crucial role in understanding the SDG mapping at the researcher level.

## Conclusion

Benchmarking of the BRICS countries revealed the extent of work that has been done to address the societal challenges globally, and managing it through 16 SDGs is the best way to intricate that universities are the change agent of the society. The responsibilities of the universities in building a sustainable community are reflected in the research output of each country. Primarily, it highlights the existing SDG research competencies at the international level among the BRICS countries. The findings in the present study will act as the foundation for formulating possibilities for SDG implementation and will serve as an input for integrating sustainable developments into research and education at universities in any country. The Spearman’s Rank Correlation results of Indian contribution on 16 SDGs demonstrated that there is already a strong relationship between many SDGs in research programs. While interpreting these findings, we could see that the current basic research activities are not directly linked to the SDGs. Our study highlights the existence of more correlation between a few SDGs. This indicates the need for a strong foundation for successfully implementing the SDG agenda in universities’ future research. The correlation among SDGs is a positive sign for the universities to execute it properly. The India-level publication output study indicates that the positive correlations among the SDGs vastly outweigh the negative ones and suggest that we need a clear strategy in mapping the existing research to SDGs. The shreds of evidence call for a deeper investigation and demand advanced strategic planning. For this, all SDGs need to act as a system of interacting cogwheels that together move with the academic research. Therefore, policies that foster multi-sectoral and cross-goal cooperative relations between SDGs must be implemented. It will play a crucial role in implementing the SDG mapping at the researcher and university levels. Future studies can be quantifying and analyzing the 16 SDG data with various metrics and comparative study with different countries (may be BRICS). Acknowledgment analysis can be done to find out the various funding sponsors who funded the SDG-related projects and can suggest future policy implications.

## Data Availability

Yes, the data is available on request.

## Code availability

The software is an open source available in the web.
